# Hypolipidemic Roles of Casein-Derived Peptides by Regulation of Trans-Intestinal Cholesterol Excretion and Bile Acid Synthesis

**DOI:** 10.3390/nu12103058

**Published:** 2020-10-06

**Authors:** Sungmin Lee, BuHyun Youn

**Affiliations:** 1 Nuclear Science Research Institute, Pusan National University, Busan 46241, Korea; smlee1048@gmail.com; 2 Department of Integrated Biological Science, Pusan National University, Busan 46241, Korea; 3 Department of Biological Sciences, Pusan National University, Busan 46241, Korea

**Keywords:** transintestinal cholesterol excretion, casein, hyperlipidemia, bioactive peptide

## Abstract

Hyperlipidemia, a syndrome characterized by an abnormal elevation of blood lipids, causes chronic lethal metabolic disorders. Although statins are regularly prescribed to patients, an alternative to treat the burden of excessive lipids is required for cholesterol control. In this study, it was found that the treatment of casein hydrolyzed by pepsin and trypsin induced trans-intestinal cholesterol excretion (TICE) through ATP-binding cassette subfamily G members 5 (ABCG5) expression. Next, we analyzed sequences of the peptides responsible for TICE induction, synthesized artificial peptides based on the sequences, and the hypolipidemic effects of the peptide treatments were assessed in both in vitro and in vivo models. We determined that two bioactive peptides contained in casein hydrolysates (SQSKVLPVPQK and HPHPHLSF) induced TICE through the expression of ABCG5 in enterocytes and suppressed hepatic mRNA expression of cytochrome P450 family 7 subfamily A member 1 (CYP7A1) and CYP8B1by ileal FGF19 expression both in an liver X receptor α (LXRα)-mediated manner. In the hyperlipidemic mouse models, the oral administration of peptides reduced serum cholesterol levels through elevation of the ABCG5 expression in proximal intestine and fecal cholesterol secretion. Besides this, peptides induced ileal expression of fibroblast growth factor 15/19 (FGF15/19) and inhibited hepatic bile acid synthesis. We found that the oral treatment of casein-derived bioactive peptides could improve hyperlipidemia by regulating intestinal excretion and hepatic synthesis of cholesterols.

## 1. Introduction

Hyperlipidemia, an abnormal elevation of lipids such as triglyceride, cholesterol, and low-density lipoprotein (LDL) in the blood, is a global metabolic syndrome. Although the causes of hyperlipidemia remain elusive, some autosomal mutations in genes involved in lipoprotein catabolism, including LDL receptor (LDLR), apolipoprotein B, proprotein convertase subtilisin/kexin type 9 (PCSK9), and upstream transcription factor 1, are reportedly related with primary hyperlipidemia [1]. In patients with hyperlipidemia, LDL clearance and LDLR expression are often inactive and hepatic lipid accumulation is elevated, further inducing blood cholesterol accumulation through hepatic very-low-density lipoprotein production [2]. Additionally, hyperlipidemia is an important risk factor leading to secondary metabolic diseases such as type 2 diabetes, obesity, and non-alcoholic fatty liver disease and cardiovascular diseases such as arteriosclerosis and myocardial infarction. In obese individuals, weight loss and an improvement in body mass index (BMI) could suppress hyperlipidemia and its related disorders; however, the need for strategies to alleviate excessive blood lipids is urgent.

As a therapy for hyperlipidemia, statins, inhibitors of 3-hydroxy-3-methylglutaryl-coenzyme A, are widely prescribed to suppress cholesterol synthesis and lower blood triglyceride and cholesterol levels, with non-statin therapies, including fibrates, omega-3-fatty acid, and niacin, often used in combination in patients presenting statin intolerance [3]. However, products of 3-hydroxy-3-methylglutaryl-coenzyme A are also utilized for essential biomaterials, and inappropriate statin prescriptions could result in statin-associated symptoms in the muscles, pancreas, and central nervous system [4,5]. Recently, a combination therapy comprised of statins and ezetimibe has demonstrated the potential to overcome limitations of statin monotherapy via the inhibition of intestinal cholesterol absorption and the increase of cholesterol excretion [6]. In addition, a PSCK9 inhibitor, a suppresser of hepatic LDLR expression, has also indicated potential in non-statin hypolipidemic therapy [7]. Nevertheless, further improvement in therapeutic strategies is imperative for the fundamental regulation of hyperlipidemia.

Although the liver is the most principal organ to synthesize and secrete cholesterol into intestinal lumen, it was supposed that approximately 95% of cholesterol excreted through hepato-biliary cholesterol transport was reabsorbed by the intestine [8]. In the early 21st century, it was demonstrated that hepato-biliary transport is not the sole pathway for secreting cholesterol; there also exists a direct transport pathway through intestinal enterocytes called trans-intestinal cholesterol secretion or excretion (TICE) [9]. Studies have reported that the amount of cholesterol transported by TICE accounts for 30–40% of the total fecal cholesterol excretion, which can be further increased by pharmacological induction [10]. Regarding the molecular mechanisms, dimerized ATP-binding cassette subfamily G members 5 and 8 (ABCG5 and 8) account for most of the cholesterol transport, along with dimerized ATP-binding cassette sub-family B 1A and B (ABCB1A and B) to a certain extent [11]. Although liver X receptor α (LXRα) that is reportedly involved in cholesterol removal has been suggested as an upstream regulator of ABCG5 and 8 expression, the TICE regulatory mechanism has not been fully understood [12]. In a recent study, the agonist of farnesoid X receptor (FXR) increases the ileal secretion of FGF15/19 and alters the bile acid profile to further enhance TICE activity [10]. Therefore, elucidating the connecting networks among the main players of the TICE is strongly warranted for the precise interpretation of this biological event.

Proteins are primarily digested by pepsin in the stomach and by peptidase in the small intestine, forming single amino acids. In a gastrojejunal kinetic study, the complete digestion of proteins in the duodenum required more than 6 h, suggesting that digestive organs can be exposed to digestive intermediates for several hours [13]. Recently, many reports have observed the significant bioactivity of protein hydrolysates during protein digestion. Their bioactivity was determined through the identification of peptide sequences, and the bioactivities were maintained even with the intake of polypeptides, which are not complete proteins [14]. Bioactive polypeptides usually consist of 2–20 amino acids and have a range of functions, including immune regulation, antioxidant, and metabolic regulation [15]. In previous studies, bioactive peptides derived from β-lactoglobulin, accounting for around 10% of milk proteins, reportedly lowered the serum cholesterol levels [16,17]. By providing basic information regarding certain peptides, these studies showed the significance of milk-derived bioactive peptides. Besides this, the hydrolysates of casein, accounting for approximately 80% of proteins contained in milk, also have been demonstrated to show antioxidant, anti-obese, and anti-hypertensive functions [18,19,20]. Although some reports determined the bioactive peptides involved in the functions, those involved in lipid metabolism regulation are still elusive.

In this study, we attempted to unveil the bioactivity and molecular mechanisms of casein hydrolysates, as well as their derivative peptides, in the regulation of blood cholesterol levels, along with the underlying molecular mechanisms utilizing synthetic peptides in both in vitro and in vivo models. Moreover, we suggested the potential of the clinical application of the casein-derived bioactive peptides as adjuvants of treatment for hyperlipidemic patients.

## 2. Materials and Methods

### 2.1. Chemicals, Antibodies, and Reagents

Bovine casein, pepsin, and trypsin for preparation of casein hydrolysate and cholesterol, monoolein, and sodium taurocholate for cholesterol solution were purchased from Sigma Aldrich (St. Louis, MO, USA). SiRNAs specific for human mRNA of FGF19 and control siRNA were purchased from Bioneer (Daejeon, Korea). Antibodies against ABCG5 and ABCG8 were purchased from Abcam (Cambridge, MA, USA) and those against FGF15, FGF19, and α-tubulin were purchased from Santa Cruz Biotechnology (Santa Cruz, CA, USA). Eagle’s Minimum Essential Medium (MEM), Dulbecco’s Modified MEM (DMEM), fetal bovine serum (FBS), penicillin, streptomycin, and TRIzol were obtained from Thermo Fisher Scientific (Cleveland, OH, USA).

### 2.2. Cell Culture and Treatment

Human colorectal cancer cell line Caco-2 and human normal hepatocyte cell line MIHA were cultured following the previous study [21]. In short, the culture medium was prepared based on MEM (for Caco-2) and DMEM (for MIHA) supplemented with 10% of FBS and 1% of penicillin and streptomycin. The cells were grown in an incubator with an atmosphere of 37 °C of temperature, 5% of CO_2_, and humidity. To assess the effects upon treatment, the cells were incubated in serum-free media 24 h before the treatment. 

For preparation of conditioned media (CM), Caco-2 cells (5 × 10^5^) were seeded in 100 mm culture dishes and treated with peptide (1 μg/mL) or/and GSK2033 (1 μM) in serum-free medium for 24 h following the previous study [22]. Subsequently, the medium was exchanged with fresh serum-free medium and collected as CM after 24 h. CM was concentrated 2-fold using a Centricon-10 concentrator (Millipore, Billerica, MA, USA) at 4 °C and filtered with 0.2 μm syringe filter. CM was treated to MIHA cells for further experiments.

### 2.3. Casein Hydrolysis

The casein solution was prepared at the concentration of 5 mg/mL. The pH was adjusted to 2 with 40% HCl solution and incubated with pepsin (0.1% or 0.4% *w/v*) at 37 °C for 2 h. Then, the pH was adjusted to 7.6 with NaOH solution and incubated with trypsin (0.1% or 0.4% *w/v*) at 37 °C for 2 h. A portion of the hydrolysates was mixed with SDS sample buffer and loaded with SDS-PAGE and stained with Coomassie Blue.

### 2.4. Total RNA Isolation and qRT-PCR

For mRNA expression assessment, qRT-PCR was performed following the previous study [23]. Briefly, RNA was isolated with TRIzol following the manufacturers’ instructions and real-time qRT-PCR was performed using an Applied Biosystems StepOne Real-Time PCR System (Applied Biosystems, Foster City, CA, USA). It was performed for 40 cycles of 95 °C for 15 s and 60 °C for 1 min followed by thermal denaturation. The sequences of the primers used are listed in Table S1. Each sample was assessed by triplication.

### 2.5. Western Blot

The protein expression was validated as previously described [24]. Briefly, whole cell lysates (WCL) were prepared using radioimmunoprecipitation assay (RIPA) lysis buffer (50 mM Tris, pH 7.4, 150 mM NaCl, 1% Triton X-100, 25 mM NaF, 1 mM dithiothreitol (DTT), and 20 mM ethyleneglycoltetraacetic acid (EGTA) supplemented with protease inhibitors) and the protein concentrations were determined using a BioRad protein assay kit (BioRad Laboratories, Hercules, CA, USA). Protein samples were subjected to SDS-PAGE, transferred to a nitrocellulose membrane and then blocked with 5% bovine serum albumin in tris-buffered saline with Tween 20 (TBST) (10 mM Tris, 100 mM NaCl, and 0.1% Tween 20). Next, membranes were probed with specific primary antibodies and subsequently peroxidase-conjugated secondary antibody (Santa Cruz Biotechnology, Santa Cruz, CA, USA). The membranes were analyzed using an ECL detection system (Roche Applied Science, Indianapolis, IN, USA) with iBright chemi-doc fl000 from Thermo Fisher Scientific.

### 2.6. Cholesterol Assay

To assess the total cholesterol levels contained in cells, media, serum, and feces, total cholesterol assay kit (Cell Biolabs, San Diego, CA, USA) was utilized. Following the manufacturers’ instructions, cells and feces were homogenized in extraction solution (mixture of chloroform:isopropanol:NP-40 (7:11:0.1)), centrifuged at 15,000× *g* for 10 min, and the supernatant was obtained. The solution was dried at 50 °C and dried lipids were dissolved in assay buffer. Media and serum were diluted in assay buffer. Then, the samples were applied to cholesterol assay and detected at 560 nm. Each sample was assessed by triplication.

### 2.7. In Vitro TICE Assay

Following the previous study, Caco-2 cells were seeded on insert of the trans-well and differentiated for 7 days [25]. To prepare a medium with cholesterol, MEM medium was supplemented with monoolein (30 μM), sodium taurocholate (500 μM), and/or cholesterol (100 μM) and subsequently sonicated for 15 min for formation of micelles. To assess the in vitro TICE, the upper chamber was filled with the medium without cholesterol and the lower chamber was filled with medium with cholesterol. The media in upper chamber was harvested after 24 h from peptides and GSK2033 treatment and applied to cholesterol assay. 

### 2.8. High-Performance Liquid Chromatography (HPLC) Analysis of Casein Hydrolysates

For separation of peptides contained in protein hydrolysates, HPLC was utilized. Waters 1525 Binary HPLC pump (Wasters, Milford, MA, USA), Waters 2489 UV/Visible detector (Waters), and Sunfire C18 column (4.6 × 250 mm) were used. The mobile phase was an isocratic combination of acetonitrile:H_2_O (50:50) with a flow rate of 1 mL/min. Following the real-time UV detection result, the elutes were respectively collected.

### 2.9. Peptide Sequencing and Synthesis

To analyze bioactive peptides contained in HPLC elutes of casein hydrolysates, the bioactive fraction was applied to peptide identification LC-MS/MS performed by Life Science Laboratory. Co. (http://www.emass.co.kr, Seoul, Korea). Following the peptide identification results, artificial peptides were synthesized by Peptron Co. (http://peptron.co.kr, Daejeon, Korea).

### 2.10. Animal Care Protocol

Six-week-old male C57BL/6 mice (Orient Bio, Seongnam, Korea) were used for the in vivo experiments following the previous study [26]. The protocols used were approved by the Institutional Animal Care and Use Committee of Pusan National University (Busan, Korea) and performed in accordance with the provisions of the NIH Guide for the Care and Use of Laboratory Animals. The mice were housed individually or in groups of up to five in sterile cages. They were maintained in animal care facilities in a temperature regulated room (23 °C ± 1 °C) with a 12 h light–dark cycle and quarantined for 1 week prior to the study. The animals had access to water and the standard mouse chow diet (20.1% crude protein, 9.6% of crude fat, 4.6% crude fiber, 5.8% ash, minerals, and vitamins) or high cholesterol diet (HCD, 19.5% of crude protein, 32.5% of sucrose, 15% of corn starch, 21% of milk fat, 1.25% of cholesterol, 0.5% of cholic acid, minerals, and vitamins) ad libitum. The animal protocol used in this study was approved by the Pusan National University Institutional Animal Care and Use Committee (PNU-IACUC) for ethical procedures and scientific care (Approval Number PNU-2017-1712, dated 10 November 2017).

Before the experiment, mice were randomly divided into each experimental group (total *n* = 50 and *n* = 10 in each group). For establishment of hyperlipidemia models and assessment of peptide effects, the mice were fed with HCD and casein hydrolysates or peptides were orally administrated by 250 or 10 mg/kg/day, respectively. At the end of administration, the mice were given last administration of casein or peptide and fasted for 6 h before sacrifice. The mice were anesthetized, perfused, and blood, liver, small intestine (proximal, distal), and feces were harvested. All lobes of perfused liver were separately harvested and used for RNA extraction. The lumen of small intestine was washed with cold PBS and 10 cm of proximal or distal intestine was harvested, respectively. Entire layer of intestine was utilized for further experiments.

### 2.11. Enzyme-Linked Immunosorbent Assay (ELISA)

To assess the secretory FGF15/19 in serum and media samples, indirect ELISA was performed. Briefly, the samples were serially attached to the 96-well immunoplates (SPL, Seoul, Korea), blocked with 1% BSA in PBS, and probed with primary antibodies and HRP-conjugated secondary antibodies. Tetramethylbenzidine (TMB) substrates were utilized and detected at 450 nm. Each sample was assessed by triplication.

### 2.12. Statistical Analysis 

All numerical data are presented as the mean ± standard error of the mean from at least three independent experiments. For quantification, data were analyzed using *t*-tests and one-way ANOVA with Tukey comparison of each column. The Prism 5 software (GraphPad Software, San Diego, CA, USA) was used for all statistical analyses. A *p*-value < 0.05 was considered statistically significant.

## 3. Results

### 3.1. Casein Hydrolysates Regulate Hyperlipidemia and Enhance TICE

**Figure 1:**
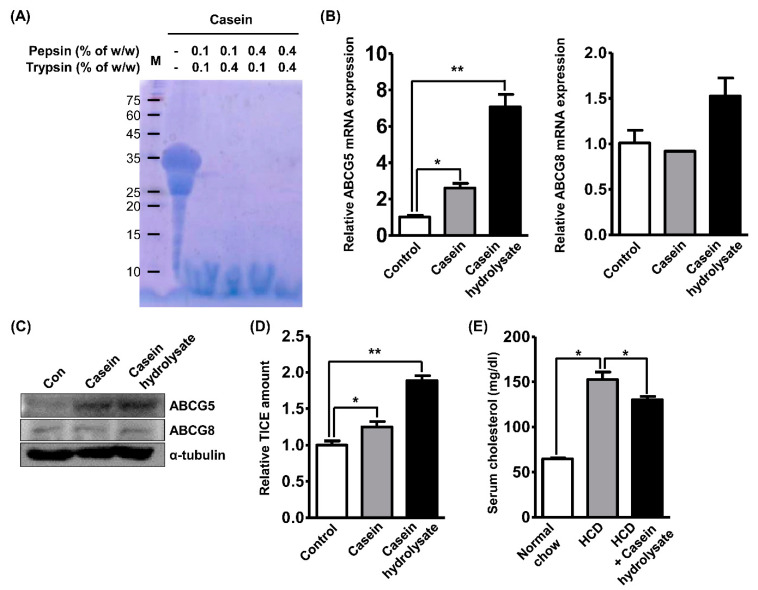
Casein hydrolysates regulate hyperlipidemia and enhance trans-intestinal cholesterol excretion (TICE). (**A**) The hydrolysis of the casein by pepsin and trypsin was validated by Coomassie Blue staining. Pepsin and trypsin were treated at a concentration as described and optimal enzymatic environment. (**B**,**C**) The mRNA or protein expression of ABCG5 and ABCG8 were assessed by qRT-PCR or Western blot analysis, respectively, in Caco-2 cells treated with casein or casein hydrolysates (2 mg/mL). (**D**) The amount of in vitro TICE was assessed using trans-well chamber and cholesterol assay following casein and casein hydrolysate treatment. (**E**) Alterations of serum cholesterol level of mice fed with high-cholesterol diet (HCD) or/and casein hydrolysates (250 mg/kg/day) were assessed by cholesterol assay. * *p* < 0.05, ** *p* < 0.01.

Reportedly, the bioactivity of casein is mediated by hydrolysis in the digestive system [18]. To validate the role of digestive casein, we prepared casein hydrolysates using 5% *w/v* solution of purified casein (a mixture of α-s1, α-s2, β-, and κ-casein). The casein solution was serially incubated with pepsin and trypsin under the optimal enzymatic conditions and digestion was verified through SDS-PAGE and Coomassie Blue staining (Figure 1A). Four conditions successfully digested casein protein and only small peptides were detected. To investigate the role of casein hydrolysate in TICE induction, we adopted the Caco-2 cell line as an in vitro model of the small intestine, as previously described [25]. Cells were treated with casein protein or casein hydrolysates at a concentration of 2 mg/mL and the mRNA expressions of ABCG5 and ABCG8 were assessed following the previous study [27]. In Figure 1B, we observed that the treatment of both casein and casein hydrolysate increased the mRNA expression of ABCG5 (2.5- or 7-fold, respectively) and only casein hydrolysate treatment significantly increased the mRNA expression of ABCG8 (1.5-fold). Furthermore, the protein expression of ABCG5 and ABCG8 was assessed using Western blot and it was found that casein treatment increased ABCG5 expression and casein hydrolysates further increased this expression (Figure 1C). To assess the effects of casein hydrolysates on cholesterol regulation, we performed an in vitro TICE assay using polarized Caco-2 cells in a trans-well chamber. As shown in Figure 1D, casein treatment increased the amount of topical cholesterol transported via TICE by 20% and casein hydrolysate treatment increased it by 80%. Next, we performed experiments utilizing the hyperlipidemia mouse model fed with a high-cholesterol diet (HCD) to validate the in vivo hypolipidemic effects of the casein hydrolysates. Based on the previous study, we daily administered casein hydrolysates (250 mg/kg/day) to mice fed with HCD for 3 weeks and observed that the serum cholesterol level was reduced by 13% compared with that of mice fed with the HCD alone (Figure 1E) [28]. Regarding these results about both the in vitro and in vivo hypolipidemic effects of casein hydrolysates, we hypothesized that the induction of TICE in enterocytes is involved in the effects and conducted further investigations.

### 3.2. TICE Is Induced by Bioactive Peptides in Casein Hydrolysates

**Figure 2:**
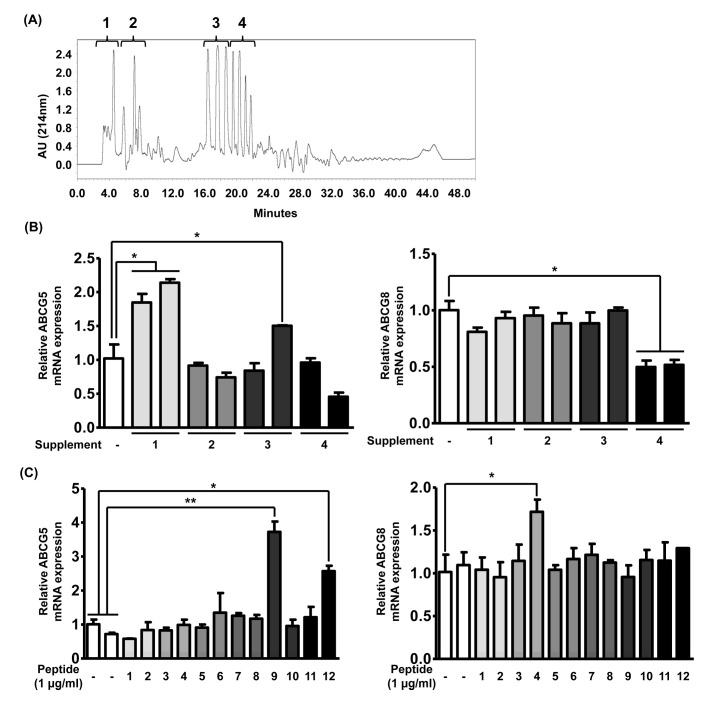
TICE is induced by bioactive peptide in casein hydrolysates. (**A**) A graph showing results of HPLC analysis. The proportion of acetonitrile in flowing solvent gradually increased from 0% to 50% for 50 min. The elute was collected into four separates and they were numbered following the order. (**B**) The alteration of mRNA expression of ABCG5 and ABCG8 was assessed by qRT-PCR in Caco-2 cells treated with each HPLC elute from 2 mg of casein hydrolysates. (**C**) The alteration of mRNA expression of ABCG5 and ABCG8 was assessed by qRT-PCR in Caco-2 cells following treatment of 12 synthetic peptides derived from HPLC elute #1. * *p* < 0.05, ** *p* < 0.01.

Reportedly, the intake of the specific proteins or peptides called bioactive peptides mediates biological effects. Although the molecular mechanisms of the action of bioactive peptides have not been fully elucidated, previous studies have suggested that the effects of these peptides are determined by their sequences [29]. Based on these findings, we hypothesized that the hypolipidemic effects of casein and casein hydrolysates are mediated by bioactive peptides that can be generated by casein digestion. Utilizing HPLC, we separated casein hydrolysates into four fractions based on their hydrophobicity (Figure 2A). To investigate the effects of individual fractions, each fraction was administrated to Caco-2 cells, and the mRNA expressions of ABCG5 and ABCG8 were assessed. As shown in Figure 2B, we observed that only fraction #1 demonstrated an increase in the mRNA expression of ABCG5 by 2-fold and none of the fractions showed a significant increase in that of ABCG8. Next, we analyzed the peptide sequences in fraction #1 through peptide identification to determine those increasing ABCG5 mRNA expression. As listed in Table 1, we observed 12 peptide sequences in fraction #1 and numbered each peptide. To verify the effects of each of these peptides, we prepared 12 synthetic peptides and administrated each peptide to Caco-2 cells at a concentration of 1 μg/mL following the previous study [29].

As shown in Figure 2C, we observed that peptides 9 and 12 significantly increased ABCG5 mRNA expression by 5- or 2.5-fold, respectively, and peptide 4 showed significantly increased ABCG8 mRNA expression by 1.8-fold. Although fraction #1 only elevated the ABCG5 expression, we selected peptides 4, 9, and 12 as promising regulators of TICE and utilized these for further experiments. Given these results, we validated the notion that the bioactivity of the protein hydrolysates can be attributed to specific bioactive peptides contained in them.

### 3.3. Bioactive Peptides Enhance TICE through Activation of LXRα

**Figure 3:**
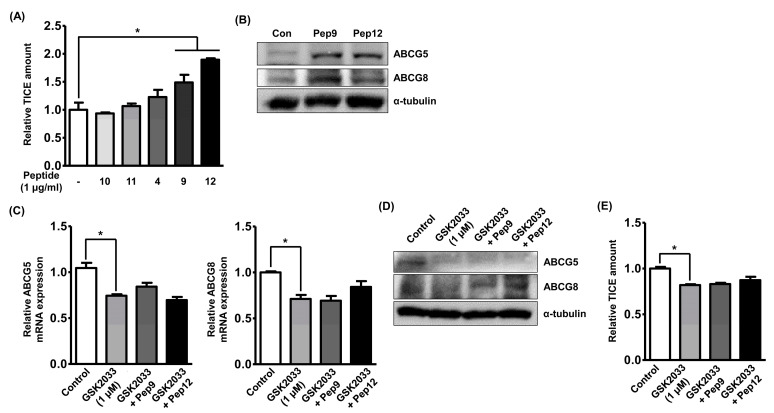
Bioactive peptide enhances TICE through activation of LXRα. (**A**) The amount of in vitro TICE was assessed by cholesterol assay in Caco-2 cells following treatment of synthetic peptides. (**B**) The alterations of protein expression of ABCG5 and ABCG8 were assessed by Western blot analysis following treatment of peptide 9 or 12. (**C**,**D**) The alterations of mRNA and protein expression of ABCG5 and ABCG8 were assessed by qRT-PCR or Western blot analysis, respectively, in Caco-2 cells following treatment of GSK2033 at a concentration of 1 μM and peptide 9 or 12. (**E**) The amount of in vitro TICE was assessed by cholesterol assay in Caco-2 cells following treatment of GSK2033 at a concentration of 1 μM and peptide 9 or 12. * *p* < 0.05.

To validate the role of peptides in the induction of TICE, we assessed the in vitro TICE amount in Caco-2 cells treated with each identified peptide. Because the bioactivities of peptides 6, 10, and 11 have not been reported and peptides 10 and 11 showed similar effects to those of non-treated cells, peptides 10 and 11 were administered to Caco-2 cells as negative controls, and we observed that only peptides 9 and 12 could increase in vitro TICE. As the TICE was not significantly induced by peptide 4, peptides 9 and 12 were utilized in further experiments. To validate the alteration of ABCG5 and ABCG8 protein expression levels following peptide treatments, we performed a Western blot analysis using Caco-2 cells. As shown in Figure 3B, we observed that the peptide treatments increased the expression of both ABCG5 and ABCG8, which was not in agreement with the qRT-PCR results. Next, we investigated the molecular pathways activated by the peptide treatments to induce ABCG5 expression in the intestine. Although the upstream regulators of ABCG5 and ABCG8 expression are not fully understood, a previous study has suggested that active LXRα is primarily involved in the transcription of the *ABCG5* gene [30]. Next, GSK2033, a specific LXRα antagonist, was treated at a concentration of 1 μg/mL following the previous study, and it reduced the mRNA expression of ABCG5 and ABCG8 by 25% in Caco-2 cells while also diminishing the effects of the peptide treatments (Figure 3C) [31]. GSK2033 treatment decreased the protein expression of ABCG5, but not that of ABCG8, indicating the involvement of independent molecular mechanisms to maintain ABCG8 expression (Figure 3D). Furthermore, the amount of in vitro TICE was reduced following GSK2033 treatment by 20%, which was similar to changes in ABCG5 and ABCG8 mRNA expression even in the presence of peptides (Figure 3E). Based on these results, we inferred that casein-derived peptides 9 and 12 induced TICE through LXRα-dependent ABCG5 expression.

### 3.4. Enterocyte-Derived FGF19 Alters the Bile Acid Synthesis in the Liver

**Figure 4:**
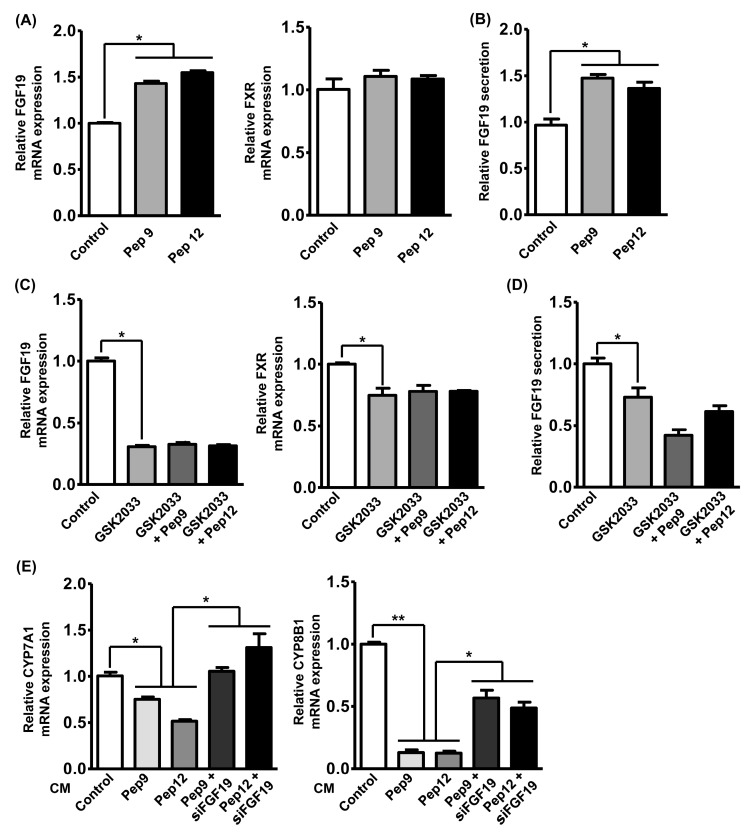
Enterocyte-derived FGF19 alters bile acid synthesis in the liver. (**A**) The alteration of mRNA expression of FGF19 and FXR was assessed by qRT-PCR in Caco-2 cells following treatment with peptide 9 or 12. (**B**) The alteration of secretory FGF19 level in conditioned media (CM) of Caco-2 cells was assessed by ELISA following treatment with peptide 9 or 12. (**C**) The alteration of mRNA expression of FGF19 and FXR was assessed by qRT-PCR in Caco-2 cells following treatment of GSK2033 and peptide 9 or 12. (**D**) The alteration of secretory FGF19 level in CM of Caco-2 cells was assessed by ELISA following treatment with GSK2033 and peptide 9 or 12. (**E**) The alteration of mRNA expression of CYP7A1 and CYP8B1 was assessed by qRT-PCR in MIHA cells following treatment with 4x concentrated CM from Caco-2 cells treated with peptide 9 or 12 and siRNA against mRNA of FGF19. * *p* < 0.05, ** *p* < 0.01.

Although TICE accounts for one-third of fecal cholesterol excretion, hepato-biliary cholesterol transport plays an important role in fecal cholesterol excretion and regulation of hyperlipidemia [11]. In a previous study, the in vivo TICE amount could be further increased by modulating the intestinal bile acid profiles, which are controlled by hepatic bile acid metabolism [10]. Hepatic bile acid metabolism is reportedly regulated by secretory proteins from intestinal tissues, which forms the hepatic–intestinal bile acid regulatory cycle. In the present study, the expression of fibroblast growth factor 19 (FGF19), a representative intestinal secretory factor regulating hepatic bile acid metabolism, was assessed following peptide treatment. Additionally, regulation of intestinal FXR expression is a determinant of FGF19 expression and TICE, so we also assessed its expression following peptide treatments. As shown in Figure 4A, treatment with peptides 9 and 12 elevated FGF19 mRNA expression by 1.5-fold, while the expression of the FXR was unaltered following peptide treatment. The increased FGF19 expression also resulted in the 1.5-fold of elevation of FGF19 secretion in the cultured medium (Figure 4B). In a previous study, LXRα ligand increased intestinal expression of FGF19 and the bioactivities of peptides 9 and 12 were mediated by the LXRα signaling pathway (Figure 3); therefore, we also assessed the alteration of FGF19 and FXR expression following the GSK2033 and peptide treatments [32]. As shown in Figure 4C, we observed that GSK2033 treatment significantly suppressed mRNA expressions of FGF19 and FXR and peptide treatments did not rescue their expressions. Furthermore, we observed that FGF19 secretion was suppressed by GSK2033 and not recovered by peptide treatment (Figure 4D). To validate the role of peptide-induced FGF19 in the regulation of hepatic bile acid metabolism, we administrated CM from Caco-2 cells treated with each peptide to the human normal hepatocyte cell line MIHA and assessed the expression of two major cholesterol synthesis-related genes, Cytochrome P450 family 7 subfamily A member 1 (*CYP7A1*) and *CYP8B1*. Although these two genes were decreased in the hyperlipidemic models, a recent study has reported that ileal FGF19 secretion decreased their expression and enhanced TICE by modulating bile acid synthesis [10]. Figure 4E shows that peptide-treated CM suppressed the mRNA expressions of both CYP7A1 and CYP8B1. To validate the involvement of FGF19 in hepatic CYP7A1 and CYP8B1 expression, CMs were collected from Caco-2 cells deficient in FGF19 expression by siRNA. We observed that knockdown of FGF19 in Caco-2 cells prevented the peptide-induced suppression of CYP7A1 and CYP8B1 expression in hepatocytes. Additionally, the expression changes were more drastic in CYP8B1 following either peptide or FGF19 knockdown. Collectively, we observed that casein-derived peptides could modulate not only TICE but also hepatic bile acid metabolism through FGF19 secretion.

### 3.5. Oral Administration of Bioactive Peptide Regulates In Vivo Hyperlipidemia

**Figure 5:**
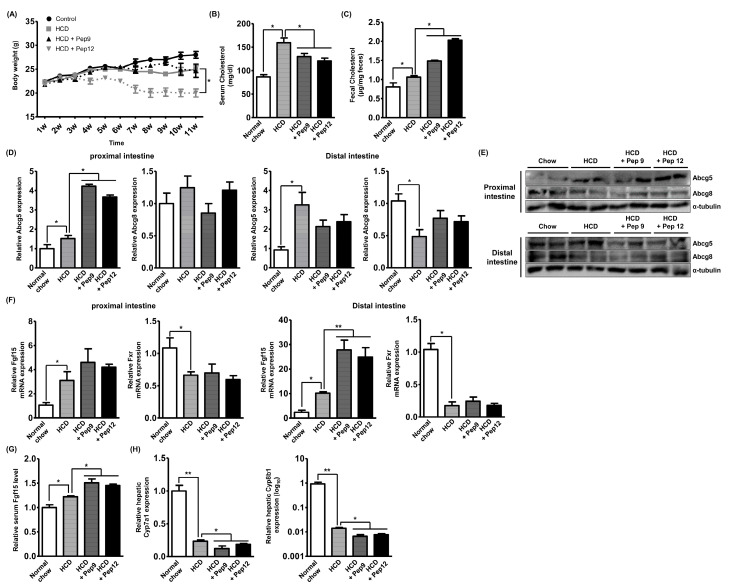
Oral administration of bioactive peptide regulates in vivo hyperlipidemia. (**A**) The change in body weight was traced after beginning of HCD and peptide administration. The treatment was started at 1st week and mice were sacrificed at 11th week after 6 h of fasting from last treatment. (**B**) Serum cholesterol levels from mice fed with HCD or/and peptides were assessed by cholesterol assay. (**C**) Fecal cholesterol levels from mice fed with HCD or/and peptides were assessed by cholesterol assay. (**D**,**E**) The mRNA and protein expression of ABCG5 and ABCG8 in proximal or distal intestine of mice fed with HCD or/and peptides were assessed by qRT-PCR or Western blot analysis. (**F**) The mRNA expression of FGF15 and FXR in proximal or distal intestine of mice fed with HCD or/and peptides was assessed by qRT-PCR. (**G**) The serum FGF15 level of mice fed with HCD or/and peptides was assessed by ELISA. (**H**) The hepatic mRNA expression of CYP7A1 and CYP8B1 of mice fed with HCD or/and peptides was assessed by qRT-PCR. * *p* < 0.05. ** *p* < 0.01.

To assess the in vivo effects of the casein-derived peptides, we established the HCD-induced hyperlipidemic mouse model and orally administered peptide 9 or 12 at a concentration of 10 mg/kg to the mice five times a week following the previous study [33]. Peptide treatment was begun concurrently with HCD administration to investigate both its prophylactic and therapeutic effects. Peptide 12 treatment decreased the body weight by 20% after 10 weeks of administration, while peptide 9 treatment failed to demonstrate this effect (Figure 5A). Regarding the hypolipidemic effects, both peptide treatments were found to diminish serum cholesterol by around 15% after 10 weeks of administration (Figure 5B). Furthermore, both peptide treatments increased the amount of fecal cholesterol by more than 50% (Figure 5C). Next, we validated the molecular events occurring in the intestine and liver after peptide administration. Although dietary peptides travel through the entire intestine, TICE reportedly occurred in the proximal intestine, near the stomach, and we thus analyzed mRNA expression of ABCG5 and ABCG8 in the proximal and distal intestine, respectively [9]. In Figure 5D, the HCD diet marginally but significantly increased only ABCG5 expression, and this expression was further increased by 3-fold following the administrations of both peptides. In the distal intestine, HCD increased the mRNA expression of ABCG5 and decreased that of ABCG8, while neither peptide treatment altered the expression levels of these genes. As shown in Figure 5E, the Western blot analysis indicated that the alteration of ABCG5 expression was correlated with the alteration in expression levels observed in qRT-PCR. Next, after the results shown in Figure 4A, we assessed the intestinal expression of FGF15 (murine homologous of *FGF19*) and FXR. As shown in Figure 5F, we observed that FGF15 mRNA expression was increased by HCD, while it was not significantly altered by peptide treatments in the proximal intestine. In the distal intestine, HCD increased FGF15 mRNA expression by 10-fold, which was markedly increased following peptide treatment by 2.5-fold compared with that of HCD group. The expression of FXR was reduced by HCD and peptide treatments failed to alter it in both parts of the intestine, which was consistent with results obtained using the Caco-2 cells. In addition, the serum FGF15 level was increased in HCD mice rather than in the controls and the level was further increased by peptide treatment (Figure 5G). This result demonstrated that the intestinal expression of FGF15 may lead to the upregulation of systemic FGF15 circulation. Finally, to validate the role of increased FGF15 in hepatic bile acid metabolism, we assessed hepatic CYP7A1 and CYP8B1 mRNA expression. As shown in Figure 5H, HCD predominantly reduced the expression of the mRNA expressions of CYP7A1 or CYP8B1 by 0.2- or 0.01-fold, respectively, which further decreased their expression by the peptide treatments. Although the degree of the downregulated expression induced by peptide treatment was little, it was shown to be statistically significant. Collectively, peptides 9 and 12 showed cholesterol-lowering effects in the hyperlipidemic mouse models through the induction of ABCG5 expression in the proximal intestine, increasing TICE and fecal cholesterol excretion. Additionally, peptides 9 and 12 induced FGF15 secretion in the intestine, further downregulating HCD-induced hepatic CYP7A1 and CYP8B1 mRNA expression, which modulates bile acid biosynthesis.

## 4. Discussion

**Figure 6:**
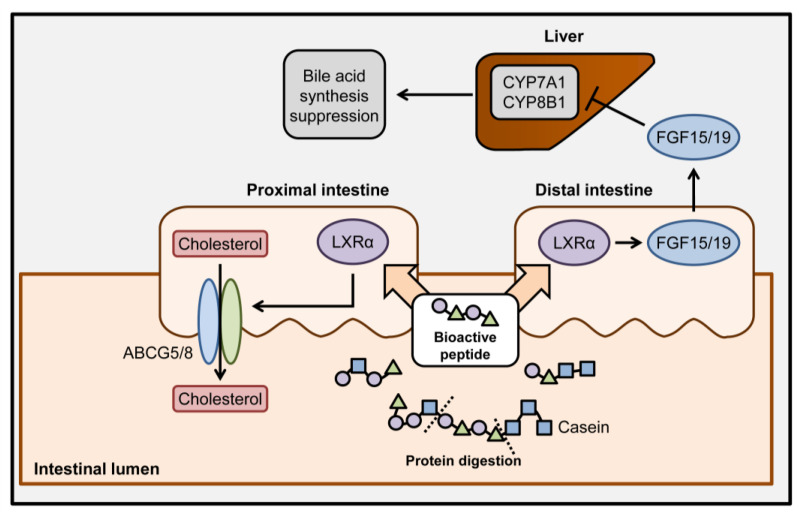
Schematic summary of hypolipidemic effects of casein-derived bioactive peptides. After dietary uptake of casein, the generation of bioactive peptides and its related biological events were depicted. Bioactive peptides derived from the enzymatic digestion of casein enhance ABCG5/8-mediated TICE in proximal intestine and increase FGF15/19 secretion through LXRα signaling activation. Ileal FGF15/19 circulates and suppresses the hepatic expression of CYP7A1 and CYP8B1, resulting in the suppression of hepatic bile acid synthesis.

As patients with hyperlipidemia and its related complications have increased globally, hyperlipidemia has become a major problem that needs to be resolved through innovative clinical research. Recently, the induction of TICE has been suggested as a promising therapeutic strategy that could be combined with another hyperlipidemic treatment [10]. In this study, we suggested two specific casein-derived peptides (SQSKVLPVPQK for peptide 9 and HPHPHLSF for peptide 12) that could induce TICE through the activation of the LXRα signaling pathway and the induction of ABCG5 expression (Figure 6). Furthermore, FGF15/19 secretion from enterocytes was induced by the peptides, which decreased hepatic bile acid synthesis to alter the hepato-biliary cholesterol homeostasis. These results suggest that dietary proteins demonstrate bioactivity through the generation of peptides during digestive hydrolysis and may be involved in the maintenance of systemic cholesterol homeostasis.

TICE has been suggested as an alternative cholesterol-regulating event of hepato-biliary cholesterol excretion. Around one-third of cholesterol excretion was mediated by TICE and the clinical potential of pharmacological induction of TICE was warranted [11]. In this study, we found that dietary peptides could induce TICE in both in vitro and in vivo models by elevating the expression of ABCG5, but not that of ABCG8. Utilizing GSK2033, we also observed that the peptide-induced ABCG5 expression was mediated by the transcriptional activity of LXRα. As the involvement of LXRα in the regulation of ABCG5 expression and induction of TICE has been previously reported, we additionally observed that the signal transduction induced by the dietary intake of bioactive peptides was coupled with the regulation of LXRα activity [30]. Although the role of intestinal ABCG5- or ABCG8-only overexpression in TICE induction has not been reported, the elevation of ABCG5 and ABCG8 expression increased TICE [34]. Additionally, with heterodimerization of ABCG5 and ABCG8 occurring in the endoplasmic reticulum and transport of them to the cellular membrane for functioning, an increase in protein transcription and translation could induce frequent physical binding [30]. The results obtained using the in vivo models showed that the peptide treatments increased ABCG5 expression only in the proximal intestine and not in the distal intestine. Previously, it has been validated that TICE primarily occurs in the proximal intestine and gradually diminishes upon movement towards the distal intestine; hence, the peptide-induced ABCG5 expression in the proximal intestine may play a significant role in elevated fecal cholesterol excretion, as confirmed in the present study [9]. Overall, we concluded that casein-derived bioactive peptides activated TICE in the proximal intestine via LXRα-ABCG5 expression.

Digestion of protein has been described as a serial enzymatic event that divides a protein into single amino acids to be absorbed by the small intestine and only the proportion of each amino acid is important, not the protein sequence. Although many studies have reported the bioactivity of casein hydrolysates lysed by various proteases, the molecular mechanisms have only been partially reported in the context of metabolic regulation [20,35]. In this study, we suggested the sequences of two peptides demonstrating bioactivity. Reportedly, peptide 9 is detected in casein hydrolysates involved in nervous system development and antioxidant effects [36,37]. Peptide 12 is present in casein hydrolysates known to have antioxidant, anti-hypertension, and osteoblast proliferation/differentiation effects [38,39,40]. Although the antioxidant effect is commonly observed, other peptides listed in the table (peptides 1 and 5) are also present in antioxidant casein hydrolysates, indicating that their molecular mechanisms need to be revealed [41,42]. A recent review explained that bioactive peptides can function in the lumen of small intestine levels and be transported into the bloodstream through microfold cells, exosomes, and enterocytes [43]. According to the data from the present study, the effects of hypolipidemic peptides from casein are inferred to exert their effects directly on enterocytes and be upstream regulators to modulate gene expression. However, the evidence is not fulfilled, and therefore, the deep investigation of each bioactive peptide characteristic is essential in future studies to identify novel promising bioactive peptides and elucidate the molecular mechanisms underlying their action.

As demonstrated in both in vitro and in vivo models, intestinal stimulation of peptides 9 and 12 resulted in the suppression of hepatic CYP7A1 and CYP8B1 expression through the induction of intestinal expression and secretion of FGF15/19. As CYP7A1 is a rate-limiting step for bile acid synthesis and CYP8B1 determines the synthetic flow of cholesterol in hepatocytes, regulating the expression levels of these proteins altered the amount and profile of biosynthetic cholesterol [8]. The results of the current study are consistent with reports indicating that hepatic CYP7A1 and CYP8B1 expression are lowered in diet-induced hyperlipidemia models and that FGF15/19 suppressed expression of both these genes [44,45]. Recently, the pharmacological induction of distal intestinal FGF15/19 led to hepatic bile acid metabolism and further enhanced fecal cholesterol excretion in combination with ezetimibe, a TICE inducer [10]. Furthermore, increased FGF15/19 also benefited patients with hyperlipidemia through systemic catabolic activation, including increased fatty acid oxidation and energy expenditure and decreased appetite and lipid synthesis [46]. These investigations were consistent with the results regarding the differential expression of Fgf15 between mouse proximal and distal intestines. Collectively, in addition to the direct induction of TICE, casein-derived peptides 9 and 12 have crucial roles in the regulation of systemic lipid metabolism via the ileal expression of FGF15/19 and the potential for combination treatment with casein-derived peptides and classical hypolipidemic drugs in patients is suggested.

## 5. Conclusions

In this study, we revealed two promising casein-derived bioactive peptides and elucidated the molecular mechanisms underlying their functions. As casein is a safe food ingredient and the bioactivities of peptides were confirmed using artificial synthetic peptides, the information regarding peptide sequences obtained here may be utilized in further studies for improving the efficacy of these peptides and elucidating their possible molecular mechanisms. Finally, the unveiled functions of food ingredients necessitate further investigation to discover promising and novel alternatives to existing therapies demonstrating adverse side effects.

## Figures and Tables

**Table 1:** Sequences of peptide contained in bioactive HPLC peptide.

No.	Sequences	Original Protein
1	HIQKEDVPSER	Alpha-s1 Casein
2	KKYKVPQL	Alpha-s1 Casein
3	EGIHAQQK	Alpha-s1 Casein
4	VKITVDDKHYQK	Alpha-s2 Casein
5	ITVDDKHYQK	Alpha-s2 Casein
6	KAMKPWIQPK	Alpha-s2 Casein
7	KIHPFAQTQ	Beta-Casein
8	HKEMPFPK	Beta-Casein
9	SQSKVLPVPQK	Beta-Casein
10	RFFSDKIAK	Kappa Casein
11	SNTVPAK	Kappa Casein
12	HPHPHLSF	Kappa Casein

## Supplementary Materials

The following are available online at https://www.mdpi.com/2072-6643/12/10/3058/s1, Table S1: Primers used for qRT-PCR to assess gene expression alterations.
