# Supplementary material for: Hypolipidemic Roles of Casein-Derived Peptides by Regulation of Trans-Intestinal Cholesterol Excretion and Bile Acid Synthesis
Source: Nutrients. 2020 Oct 6;12(10):3058. doi: 10.3390/nu12103058 (PMC7600240; doi:10.3390/nu12103058)
Supplement: Supplementary file 1 [file nutrients-12-03058-s001.pdf]

Supplementary file

Table 1. Primers used for qRT-PCR to assess gene expression alterations.

Gene Name	Forward Primer	Reverse Primer
Human ABCG5	5′-AGCAAGGAACGGGAAATAGA-3′	5′-CAGGAGAACACCCAGTTTAGAG-3′
Human ABCG8	5′- GATACAGCCGCCCTCTTTT-3′	5′-GCCCGTCTTCCAGTTCATAG-3′
Human FGF19	5′-AGATCAAGGCAGTCGCTCTG-3′	5′-AAAGCACAGTCTTCCTCCGA-3′
Human FXR	5′-AAAGTTGTGTAAGATTCACCAGCCT-3′	5′-GGTCGTTTACTCTCCATGACATCA-3′
Human CYP7A1	5′-GAC CAC ATC TTT GAT TTG G-3′	5′-CCGTTTGCCTTCTCCTAA-3′
Human CYP8B	5′-GCCTGTCCTTTGTAATGCTGA-3′	5′-GAAGCGAAAGAGGCTGTCC-3′
Human GAPDH	5′-ATGACATCAAGAAGGTGGTG-3′	5′-CATACCAGGAAATGAGCTTG-3′
Mouse Abcg5	5′-CTTCGACAAAATTGCCATCC-3′	5′-GAAAGGAACCGTGGGTAAGG-3′
Mouse Abcg8	5′- TGGTCAGTCCAACACTCTGG -3′	5′-ACTGGGTTGCCCATTTATCC-3′
Mouse Fgf15	5′-GAGGACCAAAACGAACGAAATT-3′	5′-ACGTCCTTGATGGCAATCG-3′
Mouse Fxr	5′-AAATGAGGGCTGCAAAGGTTTCT-3′	5′-TGCCCCCGTTCTTACACTTG-3′
Mouse Cyp7a1	5′-TACAGAGTGCTGGCCAAGAG-3′	5′-GCTGTCCGGATATTCAAGGA-3′
Mouse Cyp8b1	5′-CCTCTGGACAAGGGTTTTGTG-3′	5′-GCACCGTGAAGACATCCCC-3′
Mouse Gapdh	5′-CGACTTCAACAGCAACTCCCACTCTTCC-3′	5′-TGGGTGGTCCAGGGTTTCTTACTCCTT-3′
